# Incidence and risk factors for early pregnancy loss in women with first pregnancy undergoing in vitro fertilization-embryo transfer

**DOI:** 10.1186/s12884-022-04904-8

**Published:** 2022-07-19

**Authors:** Mohan Wang, Xiao Yang, Linlin Li, Haibo Zhu, Hongguo Zhang, Yuting Jiang, Ruizhi Liu

**Affiliations:** Center of Reproductive Medicine and Center of Prenatal Diagnosis, the First Hospital, Jilin University, 71 Xinmin Street, Changchun, Jilin, 130021 China

**Keywords:** Early pregnancy loss, In vitro fertilization, Infertility, Miscarriage

## Abstract

**Background:**

This study aimed to explore the incidence and influencing factors for early pregnancy loss (EPL) in infertility patients with first pregnancy undergoing in vitro fertilization (IVF) embryo transfer cycles in Jilin province, China.

**Methods:**

A retrospective study of 2709 first pregnancy embryo transfer cycles collected from January 2016 to January 2021 was performed. The cycles were divided into the EPL group (*n* = 411) and the live birth group (*n* = 2298) according to the cycle outcomes.

**Results:**

The EPL rate of the first-time pregnancies for infertility patients undergoing fresh/frozen-thaw embryo transfer cycle was 14.1%. Female patients aged 40 and older had increased odds of EPL compared to those under 35 (*OR* = 3.97, *95%CI*: 2.80–7.55). Female patients with a body mass index (BMI) of 25 or greater were more likely to have EPLs than those in the normal BMI range (*OR* = 1.32, *95%CI*: 1.03–1.70). The odds of EPL after frozen-thaw embryo transfer were higher than those after fresh embryo transfer (*OR* = 1.59, *95%CI*: 1.11–2.27). A thin endometrium on the day of embryo transfer increased the odds of EPL (*OR* = 1.84, *95%CI*: 1.01–3.33). Transferring more than two embryos had lower odds of EPL than single-embryo transfer (*OR* = 0.67, *95%CI*: 0.50–0.90). Compared with other infertility diagnoses, tubal factor alone was associated with lower odds of EPL (*OR* = 0.72, *95%CI*: 0.53–0.98).

**Conclusions:**

The risk factors for EPL were age 40 and older, obesity, frozen-thaw cycle, thin endometrium, and non-isolated tubal factor.

## Background

Infertility has gradually become a global health issue, affecting 186 million individuals worldwide [1]. In order to solve the fertility problem in infertile patients, assisted reproductive technology (ART) is increasingly used. Although ART has improved the clinical pregnancy rate, the risk of pregnancy loss is not lower than that of spontaneous conception [2]. Early pregnancy loss (EPL) commonly occurs in the first trimester of pregnancy and accounts for 80% of pregnancy loss [3, 4]. It can be traumatizing physically and psychologically and is more evident in women undergoing ART treatment [5–7]. EPL is influenced by many factors [8]. For assisted reproductive outcomes, advanced maternal age was confirmed as a powerful predictor of EPL [9, 10]. In addition to this, obesity was also generally recognized as an independent risk factor for EPL [11]. Subsequent research showed that uterine factors could predict EPL risk in all infertility diagnoses [12]. Urinary concentrations of hydroxylated polycyclic aromatic hydrocarbons (OH-PAHs) might also predict EPL in patients undergoing ART treatment [13]. However, there were also inconsistent conclusions, such as whether the anti-Müllerian hormone (AMH) level was associated with EPL. Some researchers confirmed that low AMH level was a risk factor for EPL [10]. Nevertheless, in a study on 1383 women undergoing in vitro fertilization (IVF) cycles, researchers found that EPL was not associated with low or moderately low AMH levels [14]. Therefore, the risk factors for EPL in the embryo transfer cycles still need further exploration.

First successful pregnancy is essential for infertile patients. No studies have analysed the factors associated with EPL in women with ART treatment in their first pregnancy cycles. Moreover, there was no such study in Jilin Province, a large province in northeast China. We aimed to explore the rate of EPL and its influencing factors in the first pregnancy embryo transfer cycles in Jilin province. This study will provide referable clinical suggestions for preventing EPL in the first pregnancy for infertility patients.

## Methods

### Study subjects and design

**Fig. 1:**
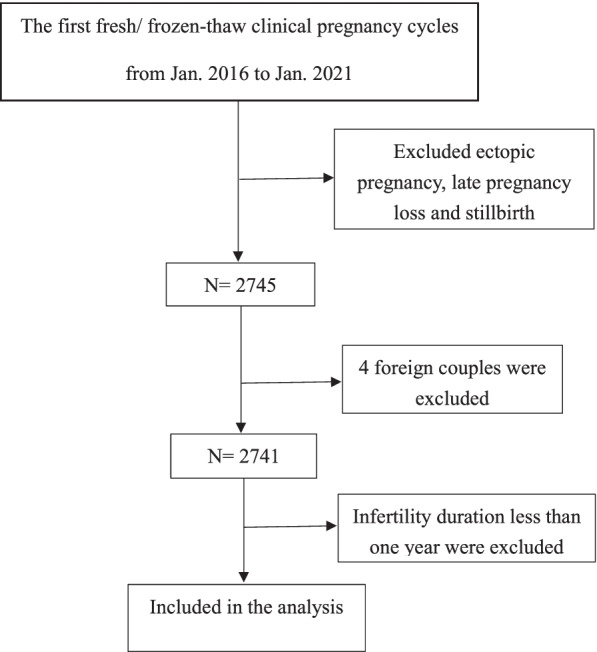
Patient inclusion flowchart

This was a retrospective study, the data were collected from electronic medical records. Patients with first clinical pregnancies in fresh or frozen-thawed IVF/intracytoplasmic sperm injection (ICSI) cycles from the Center for Reproductive Medicine, First Hospital of Jilin University (Changchun, China) between January 2016 and January 2021 were included. Sperm donation, preimplantation genetic diagnosis, and preimplantation genetic screening cycles were excluded. Figure 1 shows the patient inclusion process. After exclusions, a total of 2709 cycles were included for analysis. Two thousand seven hundred nine couples were divided into two groups according to their cycle outcomes: the EPL group and the live birth group. Other outcomes were excluded, such as ectopic pregnancy, late pregnancy loss, and stillbirth cycles. The study was approved by the ethics committee of the First Hospital of Jilin University (2021–741). Because of the retrospective character of the study, the application for exemption from informed consent was approved by the ethics committee of the First Hospital of Jilin University.

### Treatment protocol

Controlled ovarian stimulation was performed based on the gonadotropin-releasing hormone (GnRH) agonist long protocol, GnRH antagonist protocol, or other protocols, such as progestin-primed ovarian stimulation and mild stimulation. The dose of gonadotropin was adjusted according to the patients’ ovarian response. Follicle growth was monitored by transvaginal ultrasound and sex hormone tests every 2–3 days. When there were two or more dominant follicles with a diameter of ≥18 mm, recombinant human chorionic gonadotropin (hCG) was given. Oocytes were retrieved 36–38 hours after the hCG trigger. Retrieved oocytes were inseminated by IVF or ICSI according to sperm quality and clinical indications. Cleavage embryos were graded as good quality if they developed 6–9 blastomeres on Day 3, < 5% anucleate fragments, and no apparent morphologic abnormalities. On the third or fifth day, 1–3 embryos were transferred in fresh cycles. After embryo transfer, the remaining embryos were frozen. All embryos were frozen for patients with ovarian hyperstimulation syndrome (OHSS). The natural cycle, hormone replacement therapy (HRT), or downregulation HRT cycle was used for endometrial preparation for frozen-thawed embryo transfer. Luteal phase support was continued until 10 weeks of gestation if the pregnancy was achieved.

### Definitions and measurements

The variables in the analyses included patients’ sociodemographic characteristics, personal histories, infertility diagnoses, female baseline hormone levels, chromosome examinations, AMH levels, cycle type, embryo transfer status, and pregnancy outcomes. EPL was defined as the termination of pregnancy within 12 gestational weeks. Female body mass index (BMI) was divided into three groups based on the World Health Organization criteria [15]. Adverse pregnancy history referred to the experience of miscarriage, ectopic pregnancy, or stillbirth. Intrauterine insemination (IUI) unfertilized history referred to the patient’s previous experience of IUI but failure to become pregnant. The thickness of the endometrium on transfer day was divided into two groups according to a clinical practice guideline for managing thin endometrium [16].

### Data analysis

SPSS software (Version 25.0, IBM SPSS, IBM Corp, Armonk, NY, USA) was used to analyze the data. Continuous variables were tested for normality using Kolmogorov–Smirnov tests. Nonnormally distributed data are represented as the median (M) and interquartile range (Q_25_, Q_75_). Mann–Whitney U tests were conducted to investigate group differences in continuous basic characteristic variables. For categorical variables, data were compared by chi-square tests. Binary logistic regression analyses were used to identify the independent influencing factors for EPL. Pregnancy outcome was used as the dependent variable. Variables were used as independent variables, including patients’ sociodemographic characteristics, personal histories, infertility diagnoses, chromosome examinations, AMH levels, cycle type and embryo transfer status. Univariable analysis was performed for each independent variable to examine the EPL correlations. The variables that were found to be significant (female age, male age, female BMI, AMH level, cycle type, number of previous miscarriages, embryo transfer stage, number of embryos transferred, thickness of endometrium, isolated tubal factor, and isolated diminished ovarian reserve factor) were included as covariates into multivariate logistic regressions with the “enter” method to examine the independent risk factors for EPL. Odds ratios (OR) with 95% confidence intervals (CI) were used to present the model’s results. Statistical significance was set to *p* < 0.05 (two-tailed).

## Results

### Rate of EPL and basic characteristics of patients

A total of 2709 cycles were included in this study. All cycles did not include ectopic pregnancy, late pregnancy loss, or stillbirth. Four hundred eleven ended in EPL, and 2298 had live births. The EPL rate was 14.1% (411/2925). Table 1 presents the basic characteristics of the patients. Female age, male age, female BMI, luteinizing hormone (LH) levels, and distribution of cycle types showed differences (*p* < 0.05) between the two groups.

**Table 1:** The basic characteristics of patients with EPL and live birth among 2709 IVF cycles at the Center for Reproductive Medicine in Changchun, China, 2016–2021

Variables	Live birth	EPL	***P***
**Number of patients, n (%)**	2298 (84.8)	411 (15.2)	–
**Female age, M(Q**_**25**_**, Q**_**75**_**)**	31 (29, 34)	32 (29, 35)	< 0.001
**Male age, M(Q**_**25**_**, Q**_**75**_**)**	32 (30, 35)	33 (30, 37)	< 0.001
**Female ethnicity**			
Han	2066 (89.9)	374 (91.0)	0.50
Minority	232 (10.1)	37 (9.0)	
**Male ethnicity**			
Han	2097 (91.3)	366 (89.1)	0.16
Minority	201 (8.7)	45 (10.9)	
**Female education level, n (%)**			
Primary education	96 (4.2)	16 (3.9)	0.71
Secondary education	774 (33.7)	147 (35.8)	
Higher education	1428 (62.1)	248 (60.3)	
**Male education level, n (%)**			
Primary	45 (12.0)	9 (2.2)	0.52
Secondary	620 (27.0)	100 (24.3)	
Higher	1633 (71.1)	302 (73.5)	
**Female BMI, M(Q**_**25**_**, Q**_**75**_**)**	22.30 (20.08, 25.00)	22.80 (20.69, 25.81)	0.009
**Baseline hormone levels, M(Q**_**25**_**, Q**_**75**_**)**			
FSH (mIU/ml)	6.18 (5.23, 7.28)	6.08 (5.08, 7.35)	0.67
LH (mIU/ml)	4.90 (3.56, 6.73)	5.19 (3.71, 7.35)	0.034
E_2_ (pg/ml)	39.40 (29.42, 52.60)	40.30 (28.30,55.78)	0.49
**Infertility type, n (%)**			
Primary	1537 (6692)	258 (62.8)	0.11
Secondary	761 (33.1)	153 (37.2)	
**Infertility duration, M(Q**_**25**_**, Q**_**75**_**)**	3 (2, 5)	4 (2, 6)	0.08
**Infertility diagnosis, n (%)**			
Male factor	1246 (54.2)	222 (54.0)	0.60
Female factor	669 (29.1)	113 (27.5)	
Male and female factor	364 (15.8)	74 (18.0)	
Unexplained factor	19 (0.8)	2 (0.5)	
**Cycle type, n (%)**			
Fresh	416 (18.1)	47 (11.4)	0.001
Frozen-thaw	1882 (81.9)	364 (88.6)	

The continuous variables are analysed by using Mann Whitney-U test

The categorical variables are analysed by using χ^2^ test

*EPL* early pregnancy loss, *IVF* in vitro fertilization, *BMI* body mass index, *FSH* follicle stimulating hormone, *LH* luteinizing hormone, *E*_*2*_ estradiol 2

### Univariate analysis for EPL

Univariate logistic regression analyses showed that female age, male age, female BMI, AMH level, cycle type, number of previous miscarriages, embryo transfer stage, number of embryos transferred, thickness of endometrium, isolated tubal factor, and isolated diminished ovarian reserve factor were associated with EPL (*p* < 0.05). (Table 2).

**Table 2:** Univariate logistic regression analyses of different variables with EPL among first pregnancy patients in IVF cycles

Variables	EPL n %		OR	95%CI	***P***
**Female age, years**					< 0.001
< 35	289	13.5	1.00		
≧35, < 40	92	18.5	1.46	1.13–1.89	0.004
≧40	30	42.3	4.69	2.88–7.64	< 0.001
**Male age, years**					< 0.001
< 35	235	13.1	1.00		
≧35, < 40	115	17.0	1.36	1.06–1.73	0.014
≧40	61	25.3	2.24	1.63–3.09	< 0.001
**Female BMI, kg/m**^**2**^					0.13
≧18.5, < 25	251	14.3	1.00		
< 18.5	35	14.3	1.00	0.68–1.47	1.00
≧25	125	17.5	1.27	1.00–1.60	0.049
**AMH,μg/L**					0.06
≥2	302	14.4	1.00		
≥1.0, < 2	51	16.0	1.11	0.82–1.57	0.45
< 1.0	37	13.3	1.56	1.07–2.29	0.022
**Male smoking**					0.42
Never	275	14.8	1.00		
Smoking	131	15.7	1.07	0.85–1.34	0.58
Smoked in the past	5	29.4	2.39	0.84–6.85	0.10
**Cycle type**					
Fresh	47	10.2	1.00		
Frozen-thaw	364	16.2	1.71	1.24–2.36	0.001
**Adverse pregnancy history**					
No	273	14.6	1.00		
Yes	138	16.3	1.13	0.91–1.42	0.26
**Number of previous miscarriages**					0.10
0	294	14.6	1.00		
1	78	15.5	1.08	0.82–1.41	0.59
2	27	19.6	1.43	0.92–2.21	0.11
≥3	12	25.0	1.96	1.01–3.81	0.048
**IUI unfertilized history**					
No	382	15.3	1.00		
Yes	29	13.6	0.87	0.58–1.31	0.51
**IVF failed ET history**					
No	388	15.1	1.00		
Yes	23	16.0	1.07	0.67–1.69	0.78
**Female chromosome**					0.98
Normal	392	15.2	1.00		
Abnormal	18	14.9	0.98	0.56–1.63	0.93
Not examed	1	16.7	1.12	0.13–9.59	0.92
**Male chromosome**					0.77
Normal	385	15.3	1.00		
Abnormal	24	13.6	0.88	0.56–1.36	0.56
Not examed	2	14.3	0.92	0.21–4.14	0.92
**Embryo transfer stage**					
Blastocyst	168	18.3	1.00		
Cleavage stage	243	13.6	0.70	0.57–0.87	0.001
**Number of embryos transferred**					
1	122	20.8	1.00		
≥2	289	13.6	0.60	0.48–0.76	< 0.001
**Thickness of endometrium, mm**^**a**^					
≥ 7 or ≥ 8	393	14.9	1.00		
< 7 or < 8	18	24.3	1.84	1.07–3.17	0.027
**Male factor**					
No	189	15.2	1.00		
Yes	222	15.1	0.99	0.80–1.22	0.94
**Tubal factor**					
No	349	16.2	1.00		
Yes	62	11.2	0.65	0.49–0.87	0.004
**Polycystic ovarian syndrome**					
No	402	15.0	1.00		
Yes	9	25.7	1.96	0.91–4.21	0.09
**Diminished ovarian reserve**					
No	400	15.0	1.00		
Yes	11	28.9	2.31	1.14–4.70	0.020
**Ovulatory dysfunction**					
No	401	15.0	1.00		
Yes	10	26.3	1.77	0.86–3.62	0.12
**Endometriosis**					
No	407	15.1	1.00		
Yes	4	21.1	1.47	1.49–4.53	0.48

*EPL* early pregnancy loss, *IVF* in vitro fertilization, *OR* odds ratio, *CI* confidence interval, *BMI* body mass index, *AMH* anti-Müllerian hormone, *IUI* intra-uterine insemination

^a^ For fresh IVF-ET cycles, thickness of endometrium was divided into two groups with a 8 mm boundary; for frozen-thaw -ET cycles, thickness of endometrium was divided into two groups with a 7 mm boundary

### Influencing factors for EPL

Table 3 shows the results of multivariate logistic regression analysis. Female age, female BMI, cycle type, thickness of endometrium, and isolated tubal factor were independent influencing factors for EPL (*p* < 0.05). Female patients aged 40 and older had increased odds of EPL compared to those under 35 (*OR* = 3.97, *95% CI*: 2.80–7.55). Female patients with a BMI of 25 or greater were more likely to have EPLs than those in the normal BMI range (*OR* = 1.32, *95% CI*: 1.03–1.70). The odds of EPL after frozen-thaw embryo transfer were higher than those after fresh embryo transfer (*OR* = 1.59, *95% CI*: 1.11–2.27). A thin endometrium on the day of embryo transfer increased the odds of EPL (*OR* = 1.84, *95% CI*: 1.01–3.33). Transferring more than two embryos had lower odds of EPL compared to single-embryo transfer (*OR* = 0.67, *95% CI*: 0.50–0.90). Compared with other infertility diagnoses, tubal factor alone was associated with lower odds of EPL (*OR* = 0.72, *95% CI*: 0.53–0.98).

**Table 3:** Multivariate logistic regression analyses for EPL among first pregnancy patients in IVF cycles

Variables	OR	95%CI	***P***
**Female age, years**			< 0.001
< 35	1.00		
≧35, < 40	1.28	0.91–1.78	0.16
≧40	3.97	2.80–7.55	< 0.001
**Female BMI, kg/m**^**2**^			0.09
≧18.5, < 25	1.00		
< 18.5	1.07	0.71–1.59	0.76
≧25	1.32	1.03–1.70	0.029
**Cycle type**			
Fresh	1.00		
Frozen-thaw	1.59	1.11–2.27	0.011
**Thickness of endometrium, mm**^a^			
≥7/8			
< 7/8	1.84	1.01–3.33	0.046
**Number of embryos transferred**			
1	1.00		
≥2	0.67	0.50–0.90	0.008
**Tubal factor**			
No	1.00		
Yes	0.72	0.53–0.98	0.038

*EPL* early pregnancy loss, *IVF* in vitro fertilization*, OR* odds ratio*, CI* confidence interval*, BMI* body mass index

^a^ For fresh IVF-ET cycles, thickness of endometrium was divided into two groups with a 8 mm boundary; for frozen-thaw -ET cycles, thickness of endometrium was divided into two groups with a 7 mm boundary

## Discussion

The main findings of this study were as follows: the EPL rate of first-time pregnancies for infertile patients undergoing fresh/frozen-thaw embryo transfer cycles in Jilin province was 14.1%. The EPL rate was associated with female age, female BMI, cycle type, thickness of endometrium, number of embryos transferred, and isolated tubal factor.

A study based on the national ART surveillance system of the U.S. Centers for Disease Control and Prevention showed that the EPL rate in a large sample of nearly 250,000 IVF cycles was 15% [12]. The EPL rate of this study was close to this. Another large sample study of IVF embryo transfer cycles in a Chinese province found that the EPL rate was 8.9% [17]. This rate was much lower than our result. A possible reason for the difference in EPL rates was that our study only included the first clinical pregnancy cycles. In addition, sample size, treatment procedures, regional differences, and other factors also lead to the differences. Even so, we should pay attention to changes in EPL rates yearly and minimize them by controlling for risk factors.

Many studies have shown that advanced age and female obesity are risk factors for EPL, whether in spontaneous pregnancy or ART pregnancy cycles [11, 18–20]. The results of this study also confirmed these two points. Our results suggested that the odds of EPL in infertile women aged 40 or older were higher than those of women under 35. The quality of female oocytes decreases with age. This is reflected in the significant reduction of mitochondria in oocytes and may lead to abnormal chromosome meiosis, resulting in an increased risk of embryo aneuploidy [21]. This is the primary reason why age was associated with EPL. Another result showed that patients with a BMI of 25 or greater had higher odds of EPL than those within the normal BMI range. Related research indicated that 3.7% of EPLs could be avoided by keeping BMI in the normal range [18]. Obesity also reduces oocyte quality by causing inflammation in the ovaries and affecting the normal structure or function of the endometrium [22]. Therefore, more attention should be given to obese and infertile women over 40. Getting assisted reproductive treatment early or losing weight earlier may reduce the risk of EPL for infertility patients.

Evidence that frozen ET cycles increase the risk of EPL is insufficient. Previous studies have focused more on the effect of cycle type on overall pregnancy loss rates [23, 24]. Our findings suggested that the odds of EPL in frozen embryo transfer cycles were higher than those of fresh embryo transfer cycles. A large cohort study yielded a similar result [12]. However, as the conclusion that the freeze-all strategy increases clinical pregnancy rate has been gradually demonstrated, more studies are needed to demonstrate further whether frozen embryo transfers increase the EPL rate.

Endometrial thickness is one important indicator related to fertility. This was also demonstrated in this study. We found that a thin endometrium was an independent risk factor of EPL. Other studies have shown that a thin endometrial lining might increase pregnancy loss rates [17, 25]. To the best of our knowledge, this was the first study to investigate the effect of endometrial thickness on EPL rates in both fresh and frozen cycles. By exploring the relationship between the number of embryos transferred and EPL, we found that single-embryo transfer was also associated with EPL. This is consistent with a large sample study [17]. A possible explanation is that a multiple pregnancy is considered a live birth even if only one baby is delivered. However, we still need to reduce the multiple pregnancy rate by minimizing the number of transferred embryos to optimize pregnancy outcomes.

There were different conclusions regarding whether tubal factors were risk factors for EPL in infertile patients [12, 26]. In this study, patients who entered the treatment cycle solely because of tubal factors had lower odds of EPL than other factors. One possible reason is that tubal factor is a relatively mild condition among the indications for fertility treatment. Moreover, most patients in this study diagnosed with tubal factors had undergone treatment or surgical resection before entering the cycles, which might reduce the risk of EPL after pregnancies.

To the best of our knowledge, this is the first study to explore factors influencing EPL in infertile patients during their first IVF pregnancy cycles in Jilin province, China. This study analysed all relevant factors that could be collected as much as possible, hoping to find the risk factors comprehensively. The results will be helpful in clinical counselling around the risks of EPL, even if more at an information-level than at a changing-practice-level. In the future, we hope to collect data with a larger sample size from multiple centres or conduct a prospective cohort study. We also hope to include IUI cycles and other assisted reproductive treatments to draw more comprehensive and clinically valuable conclusions. However, several limitations of this study should be noted. The biggest limitation is that it is a single-centre retrospective study. Moreover, including all significant variables in univariate analyses in the multivariate regression model might result in omitting truly independent factors. Finally, because not all aborted tissues were examined, we did not conduct a detailed analysis of the causes of EPL, which might provide more valuable clinical advice.

## Conclusions

In conclusion, EPL rates could be reduced by controlling risk factors in patients undergoing their first IVF pregnancy cycles. The risk factors for EPL were age 40 or older, obesity, frozen-thaw cycle, thin endometrium, and nonisolated tubal factor. We hope that these findings will provide a reference basis for clinical treatment. More extensive and more in-depth studies are needed in the future to strengthen the conclusions.

## Data Availability

The datasets analysed during the current study are not publicly available due to the hospital policy and patients’ privacy, but are available from the corresponding author on reasonable request.

## Abbreviations

ART: Assisted reproductive technology
EPL: Early pregnancy loss
IVF: In vitro fertilization
AMH: Anti-Müllerian hormone
OH-PAHs: Hydroxylated polycyclic aromatic hydrocarbons
ICSI: Intracytoplasmic sperm injection
GnRH: Gonadotropin-releasing hormone
hCG: Human chorionic gonadotropin
OHSS: Ovarian hyperstimulation syndrome
HRT: Hormone replacement therapy
BMI: Body mass index
IUI: Intrauterine insemination
LH: Luteinizing hormone
FSH: follicle Stimulating hormone
E_2_: Estradiol 2
